# Ureteroarterial Fistulas After Robotic and Open Radical Cystectomy

**DOI:** 10.1089/cren.2015.0034

**Published:** 2016-03-01

**Authors:** Ricardo Palmerola, Mary E. Westerman, Mathew Fakhoury, Stephen A. Boorjian, Lee Richstone

**Affiliations:** ^1^ The Arthur Smith Institute for Urology–North-Shore-LIJ Health System, New Hyde Park, New York.; ^2^ Department of Urology, Mayo Clinic, Rochester, Minnesota.; ^3^ New York Institute of Technology College of Osteopathic Medicine, Old Westbury, New York.

## Abstract

Ureteroarterial fistulas (UAFs) are defined as an abnormal communication between one of the major arteries and the ureter. Urologists most frequently encounter iatrogenic fistulas occurring in patients with a history of pelvic extirpative surgery, chronic ureteral catheterization, and history of pelvic radiation. We present two cases of UAFs in patients with no history of prior radiation, who underwent open radical cystectomy and robot-assisted radical cystectomy with intracorporeal ileal conduit. Both patients developed postoperative ureteroileal anastomotic leaks that were managed with indwelling ureteral catheters. Furthermore, both patients were having left-sided UAF after presenting with nonlife threatening gross hematuria, which became brisk and pulsatile during ureteral stent exchange. Endovascular stenting was performed in both patients with resolution of hemorrhage and full recovery. In one patient, nephrostomy tubes were placed and ureteral catheters were removed; the second patient was managed with continued ureteral catheterization without further episodes of hematuria.

## Background

There are more than 100 documented cases of ureteroarterial fistulas (UAFs) in the literature with a rising incidence reported in the literature.^1^ Classically, urologists encounter UAFs when brisk, pulsatile bleeding is seen from the ureteral orifice during stent exchanges. However, patient presentation can be variable ranging from flank pain, profound hematuria with clot passage, and hemodynamic instability to intermittent hematuria. Risk factors associated with this diagnosis are chronic ureteral stenting, oncologic pelvic surgery, and pelvic radiation.^2^ However, there is a paucity of literature regarding urologic experience with UAF as a complication of radical cystectomy. Cause-specific mortality rates have declined to ∼13%, largely owing to prompt diagnosis with angiography and minimally invasive endovascular techniques to treat UAFs.^1^ Herein we present two patients to whom prolonged ureteral catheterization was necessary and who developed UAF that necessitated endovascular stenting to resolve life-threatening hemorrhage.

## Case Presentations

### Case 1

An 82-year-old gentleman without major active medical comorbidities (ASA 3, history of cerebrovascular accident) was referred for management of recurrent, high-grade nonmuscle invasive bladder cancer. Over the course of several years, he was treated with intravesical BCG, mitomycin, and gemcitabine at an outside institution. He presented with a 4 to 5 cm high-grade T1 urothelial carcinoma and concomitant carcinoma *in situ* (CIS). He had no prior history of radiation, or vascular surgery. After extensive counseling, he underwent robotic radical cystoprostatectomy with intracorporeal creation of an ileal conduit. Frozen distal ureteral margins were negative for urothelial carcinoma and CIS, the ureters were then spatulated and a Bricker ureteroileal anastomosis was performed with interrupted sutures. His final pathology analysis showed pTisN0M0 urothelial bladder cancer with 25 negative lymph nodes and pT2, Gleason 6 prostate cancer.

The immediate postoperative course was complicated by a ureteroileal anastomotic leak identified on a loopogram. This was initially managed with stomal catheter drainage and prolonged ureteral catheterization. Two months later, 7F double pigtail ureteral stents placed intraoperatively were exchanged for upside down 12F Cope loop catheters. Subsequent imaging studies revealed a persistent anastomotic leak that necessitated prolonged catheterization. Five months postoperatively, bilateral percutaneous antegrade stent exchange was necessary after resistance was met during a retrograde exchange secondary to encrusted catheters.

Approximately 8 months postcystectomy, he developed flank pain and gross hematuria with clots emanating from the ileal conduit. Upon presentation, he was febrile (101.5F) and had a leukocytosis (WBC 24). His creatinine and hemoglobin were stable from baseline (0.88 mg/dL and 13.2 g/dL, respectively). A CT of the abdomen and pelvis with contrast was unremarkable. Urine cultures obtained on admission were consistent with *Pseudomonas aeruginosa*, and blood cultures were negative. The hematuria resolved shortly after admission, and the ureteral catheters were exchanged after 1 week of antibiotics. At the time of left ureteral stent removal, brisk pulsatile bleeding became apparent from the ileal conduit. The patient became hemodynamically unstable, necessitating transfusion with eight units of packed red blood cells. Once stabilized, he underwent emergent bilateral pelvic angiography to rule out UAF; however, the study demonstrated patent iliac vessels. The patient remained stable and hematuria improved. Approximately 24 hours later, hematuria and clots developed again in the ileal conduit. A CT angiogram showed a pseudoaneurysm near the left common iliac artery, in proximity to the left ureteroileal anastomosis. The patient underwent repeat angiography that showed contrast extravasation outlining the ureter. He underwent left common iliac arteriography and deployment of a 12 × 30 mm endovascular graft. This was followed by a second 12 × 50 mm wall graft to completely seal the fistula. Completion angiography showed resolution of contrast extravasation. Once stabilized, bilateral nephrostomy tubes were placed, and 12F ureteral catheters were removed. Two months later, antegrade nephrostogram showed patent ureters bilaterally with resolution of anastomotic leak. The nephrostomy tubes were removed and his course has been uneventful at 1 year follow-up.

### Case 2

**FIG. 1.**
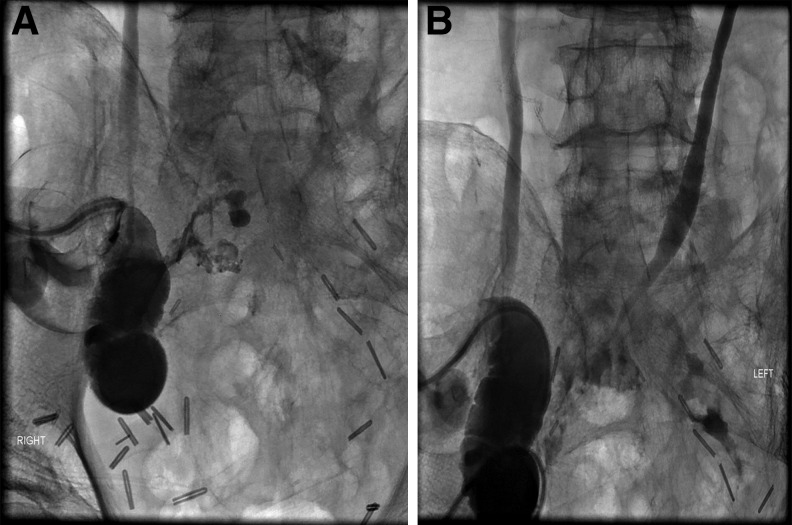
POD 21. **(A)** Loopogram showing extravasation from the ileal conduit with no contrast in the left ureter. **(B)** Antegrade left nephrostogram showing distal ureteral narrowing with anastomotic dehiscence. POD, post operative day.

An 88-year-old man (ASA 3) with stage IV chronic kidney disease presented with gross hematuria, clots, and bilateral flank pain. He was 1-year status post radical cystectomy with ileal conduit diversion for T3bN0M0 urothelial carcinoma. Intraoperatively, 7F ureteral catheters were placed after performing a Bricker ureteroileal anastomosis (as in case 1). He had never been radiated, margins at the time of surgery were negative, and he had no prior vascular surgical procedures. His postoperative course was complicated by an ileal conduit leak adjacent to the right ureteroileal anastomosis and a left ureteral conduit anastomotic dehiscence (Fig. 1), necessitating bilateral antegrade 12F ureteral stent insertion and bilateral nephrostomy tube placement by POD 30. Two months later (POD 90), the right stent and both nephrostomy tubes were removed and the left ureteral stent was exchanged as there was a persistent left ureteral leak. Approximately 3 months later, loopogram revealed a left distal ureteral stricture, which was balloon dilated and the 12F ureteral stent was exchanged.

Before his next scheduled stent exchange, the patient presented to his local emergency room with gross hematuria with clots and bilateral flank pain. He had no other systemic or constitutional complaints. He was hemodynamically stable with hemoglobin of 12.0 g/dL and stable creatinine (baseline 1.9 mg/dL). On hospital day 2, he developed worsening flank pain, and CT showed worsening hydronephrosis. Subsequently, he was taken to interventional radiology for a left retrograde stent exchange.

**FIG. 2.**
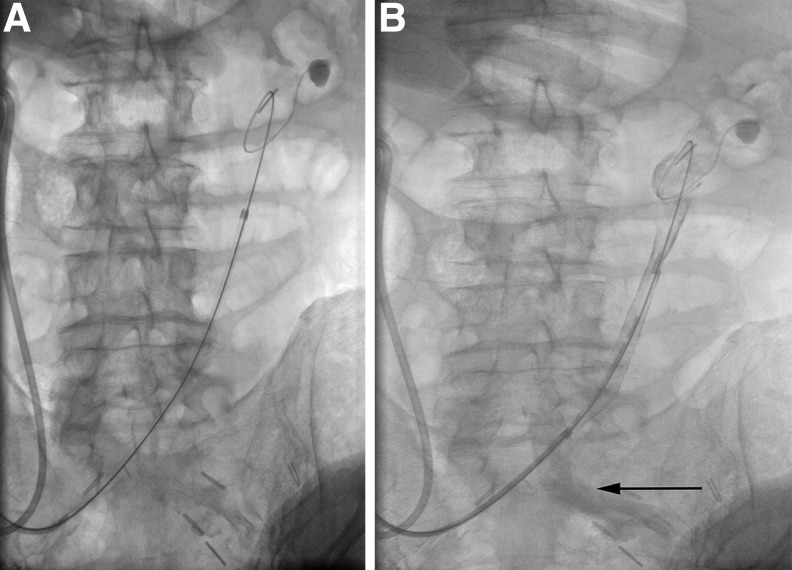
Left pullback nephrostogram showing contrast in the left common and external iliac artery. **(A)** Baseline. **(B)** Contrast in the left common iliac artery (*black arrow*).

**FIG. 3.**
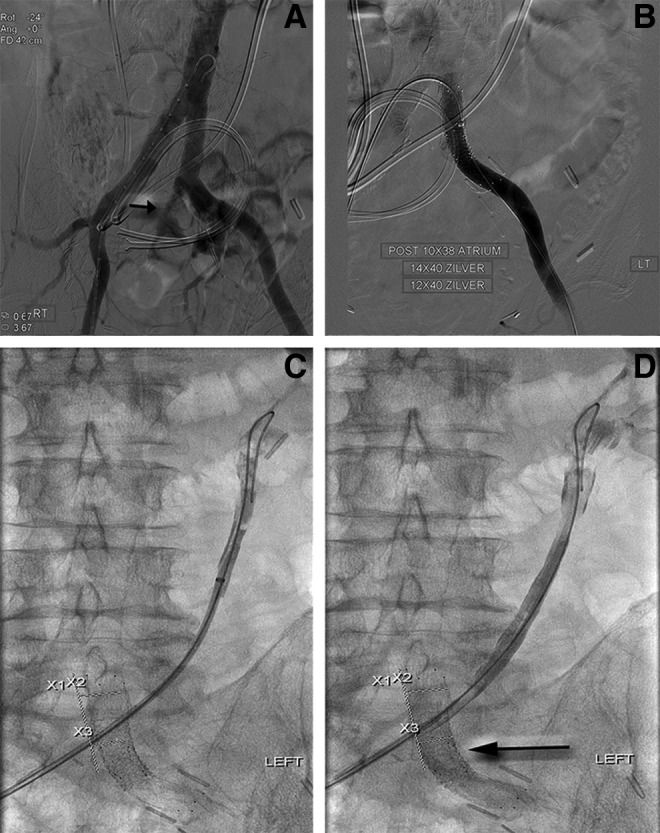
**(A)** Pelvic angiogram showing active extravasation within the left common iliac artery near the origin of the left internal iliac artery and in proximity to the left retrograde ureteral stent (*black line*). **(B)** Completion angiography after deployment of 10 × 38 mm balloon-expandable atrium stent, 14 × 40 mm and 12 × 40 mm uncovered stents at the proximal and distal ends of atrium stent showing cessation of active extravasation. **(C, D)** Pullback nephrostogram 19 days poststent placement showing persistent fistula from the left ureter into the left common iliac artery. **(A)** Contrast extravasation (*arrow*). **(B)** Completion angiography. **(C)** Scout film. **(D)** Contrast in left common iliac artery (*arrow*).

Upon removal of the left indwelling 12F stent, brisk pulsatile blood flow was noted from the ileal conduit with unstable vital signs. Left pullback nephrostogram showed a distal ureteral to the left common iliac artery fistula (Fig. 2). Pelvic angiography was emergently performed, showing active extravasation within the left common iliac artery (Fig. 3A). A 10 × 38 mm covered atrium stent was deployed at the site of extravasation. Completion angiography showed persistent filling at the site of previously noted extravasation, and two additional uncovered 14 × 40 mm and 12 × 40 mm stents were deployed at the proximal and distal ends of the atrium stent (Fig. 3B). Completion angiography showed cessation of active extravasation. Nineteen days later, the patient presented again with recurrent hemorrhage from the ileal conduit. Pullback nephrostogram of the left renal pelvis and ureter showed a persistent fistula from the left ureter to the left common iliac artery (Fig. 3C, D). Limited pelvic angiography was performed, and an additional 10 × 38 mm covered atrium stent was deployed within the left common iliac artery. The patient was subsequently discharged home with no further episodes of hematuria to date and has been undergoing stent exchanges locally.

## Discussion

The pathophysiology underlying UAF after radical cystectomy is poorly understood. One hypothesis explains that patients with a history of pelvic surgery or external beam radiation have a disruption in the vasa vasorum of the major vessels, making the arterial wall susceptible to fibrosis and fixing the ureter to the iliac vessels. After a ureteral catheter is placed, it facilitates fistula formation by transmitting pulsatile forces to the ureteral wall.^2^ Patients undergoing radical cystectomy commonly have known risk factors for UAF (see Background section). In addition to routine intraoperative ureteral catheterization, chronic ureteral catheters may be necessary in these patients as in the setting of anastomotic strictures or leaks. Although the use of smaller ureteral stents demonstrates ureteral flow rates similar to larger stents, there is a tendency to place larger diameter stents leading to increased wall compression. In our case, both patients had indwelling 12F catheters and formed UAF within 1 year of surgery, suggesting larger catheters may shorten UAF development, which has been reported to occur within 1 to 8 years of surgery.^2^

Furthermore, ureteral dissection and mobilization during cystectomy can create inadvertent ureteral injury. Devascularizing injuries and mechanical shear forces during dissection may weaken the integrity of the ureteral wall, thus increasing the susceptibility to UAF formation. One published case report described that a right UAF developed 1 month after robotic radical cystectomy and Wallace ureteroileal anastomosis. The author attributes fistula development to extensive dissection near the right external iliac arteries and liberal use of electrocautery.^3^ In our series, both fistulas were left sided and the ureteroileal anastomosis was situated in proximity to the left common iliac artery. Similarly, Van den Bergh and colleagues reviewed 139 UAFs and found that in patients undergoing urinary diversion, 63% of fistulas involved the left ureter and iliac artery; however, time to fistula development was not reported.^1^ They proposed that placing the ureteroileal anastomosis directly over the iliac artery triggered scarring, promoting fistula formation.^1^ We believe that the higher incidence of left UAF can be explained by mobilization of the left ureter to the right lower quadrant during ureteroileal anastomosis, potentially adding tension to the fragile ureteral wall. Furthermore, loss of tactile feedback during robotic surgery can limit a surgeon's ability to assess tensile forces during an intracorporeal anastomosis. Therefore, limiting indwelling ureteral catheterization, mobilization of the ureter, and replanning the site of ileal conduit anastomosis should be considered in radical cystectomy patients with the aforementioned risk factors for UAF.

Effective diagnosis and management often requires a multidisciplinary approach between urologists, vascular surgeons, and interventional radiologists. Although hematuria is frequently encountered in patients with ureteral stents, special attention should be focused on timing and severity of hematuria. Both patients presented with gross hematuria with large clot burden; however, brisk hematuria developed during stent exchange. This highlights the disruption of a tamponading thrombus during stent exchange that is typically followed by hemorrhage and potential hemodynamic instability. When UAF is strongly suspected, selective angiography should be performed emergently; however, the diagnostic yield may be limited by the presence of a thrombus occluding the fistula. In this case, provocative angiography is performed by pulling back the indwelling ureteral catheter. This increases the sensitivity by dislodging a thrombus overlying the fistula, but may provoke hemorrhage. CT angiogram may be considered, as subtle findings like an arterial pseudoaneurysm may be diagnostic of UAF. After the UAF is diagnosed, management is directed at vascular control. Contemporary management with minimally invasive endovascular stents has largely replaced open surgical repair. Although long-term data are not available, short-term results show this approach is safe and effective.^4^ Despite limitations including infection or occlusion, endovascular stents are an attractive approach for postradical cystectomy patients who may be poor repeat surgical candidates.

## Conclusion

In conclusion, UAFs are suspected in patients presenting with hematuria and risk factors including history of pelvic radiation, chronic ureteral catheterization, and pelvic surgery. A high index of suspicion is recommended for postradical cystectomy patients with new onset gross hematuria, especially in those in whom prolonged ureteral catheterization was necessary. Successful management can be achieved with endovascular stenting with or without removal of ureteral catheters.

## Abbreviations Used

BCG: Bacillus Calmette-Guerin
CIS: carcinoma *in situ*
CT: computed tomography
POD: post operative day
UAFs: ureteroarterial fistulas
